# Moral reasoning during vascular surgeons’ case conferences: finding the balance of risk and benefit by exploring the clinical details

**DOI:** 10.1007/s10730-025-09550-z

**Published:** 2025-06-25

**Authors:** Kaja Heidenreich, Marit Karlsson, Anders Bremer, Mia Svantesson

**Affiliations:** 1 University Health Care Research Centre, Faculty of Medicine and Health, Örebro University, S-huset, 2nd Floor, Örebro, 70182 SE Sweden; 2 Department of Health, Medicine and Caring Sciences, Linköping University, Linköping, 58183 SE Sweden; 3 Faculty of Health and Life Sciences, Linnaeus University, Växjö, 35195 SE Sweden

**Keywords:** Medical ethics, Physicians, Qualitative research, Vascular surgical procedures

## Background

Moral reasoning is a process of deliberating on what ought to be done by exploring what is right and wrong or virtuous and vicious (Richardson, 2014). In clinical practice, healthcare professionals practice moral reasoning by making judgments, decisions and actions when dealing with ethical issues (Beauchamp & Childress, 2013; Kaldjian, 2013). The empirical literature in clinical ethics presents a broad range of concepts attempting to capture how healthcare professionals deal with ethical issues in practice and even the term ‘ethical issue’ lacks a clear definition (Schofield et al., 2021). By employing the phenomenon moral reasoning, we intended to explore how questions of what ought to be offered to the patient were answered in a clinical context that is sensitive to medical, social and practical considerations (Musschenga, 2005).

Vascular surgery offers a range of treatments, including relieving the burden of pain and ulcers in the legs and preventing sudden death by rupture of large blood vessels (Eskandari et al., 2012). Patients who suffer from vascular diseases are often old and frail and affected by comorbidities. The surgical procedures involve risk of injury and harm, leading to ethical issues about whether surgical interventions are in the best interests of the patient (Houghton et al., 2020; Wang et al., 2018). In a recent study, we explored vascular surgeons’ moral reasoning in an out-patient setting (Heidenreich et al., 2023). Their moral reasoning comprised an exploration of what is reasonable in a quest to relieve suffering and avoid harm. This implied shifting their perspective from focusing on the vessels to considering the whole person, weighing the patient’s conflicting needs and placing responsibility for making the right decision on their own shoulders (Heidenreich et al., 2023).

In everyday practice, vascular surgeons meet in case conferences to discuss patients’ care. Case conferences for discussions about patient care are prevalent in practice and multidisciplinary team meetings (MDTs) for patients diagnosed with cancer are widely studied, with the literature describing how MDTs function as sources of giving recommendations and making final decisions as well as providing professional learning opportunities for junior medical staff (Basta et al. 2017; Walraven et al., 2022). A recent systematic review showed the impact that MDTs have on treatment decisions and supported evidence of increased survival rates for cancer patients who have been discussed in MDTs (Kočo et al., 2021).

How participants in MDTs in general deliberate ethical issues is mainly described using the concept of patient advocacy (Walraven et al., 2022). Caring for frail older patients affected by comorbidities raises ethical issues about what is in the patient’s best interest. Previous reviews of MDTs report evidence of over-treatment as well as under-treatment of these patients, which raises the question of equity (Holden et al., 2020; Stairmand et al., 2015). Additionally, previous observational studies of MDTs indicate that there are difficulties in incorporating patients’ views in the process (Salloch et al., 2014; Wihl et al., 2021). The importance of engaging in case conferences for old and frail patients with peripheral arterial disease has also been highlighted, however, without emphasizing how to deal with the ethical issues (Christie & Roditi, 2014).

In summary, how vascular surgeons deal with ethical issues by reasoning morally and clinically during case conferences are, to our knowledge, unexplored. Dealing with these issues is crucial for the quality of patient care, and further understanding may contribute to knowledge on how to adapt clinical ethics support to be relevant to the specific needs of clinicians (Rasoal et al., 2017; van Bruchem-Visser et al., 2020). Therefore, the aim of this study was to explore elements of moral reasoning during vascular surgeons’ discussions of clinical management during case conferences.

## Methods

### Design

An explorative and qualitative design was adopted, utilizing non-participant observation and individual semi-structured interviews (Patton, 2004).

### Setting

Case conferences form part of vascular surgeons regular working practices, where surgeons meet once or twice weekly to examine outpatients’ or referred patients’ x-ray images and discuss the patient’s care. In addition to surgeons, other professionals, such as radiologists, nurses, medical students, and care administrators, may also attend the conferences.

At the conferences, cases selected for discussion are referred by a surgeon or a radiologist. Most patients have been assessed by a surgeon in the outpatient clinic prior to the discussion. Some patients are discussed after a regular x-ray follow up, and some are discussed prior to a visit in the outpatient clinic.

The main purpose of the conference is to discuss the patient’s care. In some units, the surgeon who met the patient at the outpatient clinic, would later be the operating surgeon. In other units, the operating surgeon would not necessarily be the same surgeon making the decision to operate.

### Sampling and Participants

Clinics for vascular surgery in seven Swedish university hospitals were invited to participate in the research by emailing the head of each department to endorse participation. Three clinics agreed to participate, and surgeons at these clinics then received written and verbal information about the study. The first author attended seven case conferences as a non-participating observer, taking field notes, audio recording the conferences and conducting 23 follow-up interviews with surgeons who participated in the conferences by purposeful sampling. After each conference, the researcher immediately asked three or four surgeons who had been involved in the discussions during the conference about participating in a follow-up interview. Twenty-three audio-recorded follow-up interviews were conducted; twenty-one of them on the same day as the conference, two interviews the day after, and two participants were interviewed twice after two different conferences. One interview was conducted by phone, but all the others took place face-to-face, in an office or meeting room in the clinic. For demographic data relating to the participants in the follow-up interviews, see Table 1.

**Table 1:** Demographic characteristics of the participants (*n* = 21) and the follow-up interviews (*n* = 23)

Gender, *n* (%)	
Male	13 (62)
Female	8 (38)
Age, mean (range)	47 (35–69)
Years of experience, mean (range)	
In vascular surgery	14 (2–37)
Since graduation	20 (5–43)
Participants, *n*	
Hospital 1	7
Hospital 2	7
Hospital 3	7
Follow-up interviews, *n*	23
Length in minutes, mean (range)	32 (13–53)

### Data Gathering

The first author observed each case conference, listening to the discussion and taking field notes, sitting in a corner of the room outside the group without interfering with the discussion. The observer and primary coder is a PhD student and senior consultant in internal medicine and cardiology.

Spradley’s dimensions of space, actors, activities, events and time were used to guide the observations, placing an emphasis on the exploration of moral reasoning (Fangen, 2005; Spradley, 1980). This implied primarily listening carefully to how the discussion unfolded by noting how the case was presented, how the discussion of the x-ray images unfolded, which questions, comments and arguments were conveyed, and which decisions were made. The observer took field notes according to Spradley’s dimensions, emphasizing verbal utterances that seemed to be about moral reasoning. Special attention was directed to cases where engagement with and presentation of different opinions and arguments seemed to be present during the discussion.

The field notes were typed up and expanded as soon as possible after the observation and utilized to inform and guide the follow-up interviews. These interviews focused on the participants’ experiences of the conference and observations from the field notes to further explore moral reasoning. The opening question asked how the participant had experienced the conference during the morning and the interviewer thereafter utilized the field notes to further explore the previous discussion focusing on the phenomenon of moral reasoning. What the interviewer thought to be about moral reasoning was explored further during this part of the interview by asking the participants to expound about utterances noted in the fieldnotes. Questions could be composed as “I heard you say, ‘But he’s 90 years old!’ and ‘He actually has a black toe’ Could you please tell me more about that?” This explorative part of the interview generated knowledge about how normative considerations are hidden in utterances about patient’s care. At the end of the interview, the participants were also asked about the function of the conference in the surgeons’ decision-making process.

Probes further exploring their reasoning could be constructed as “Can you describe more about…?”, and “What did you mean when you said…?” Follow-up questions were adapted to the situation and could comprise medical and practical aspects, further elaborating on what they considered to be important from different perspectives and stakeholders during the discussion. A research secretary transcribed the audio-recorded conferences and follow-up interviews verbatim. All sensitive and confidential patient data obtained during the audio recording of the conferences were anonymized in the transcripts. For a description of the final dataset, see Table 2.

**Table 2:** Overview of case conferences (*n* = 7) with participating surgeons (*n* = 57 and 21)

Hospital	Conference	Length hh: mm	Patients discussed	Conference participants		Follow-up interviews
				Surgeons	Other	
1	1	01:16	12	8	3 MS, 1 RN, 1 R	3*
	2	00:36	9	6	3 MS, 1 R	3*
	3	00:25	7	7	3 MS, 1 R	3*
2	4	00:33	5	6	2 MS, 1 RN	4
	5	00:55	10	6	2 MS, 1 RN	3
3	6	01:10	13	12	1 RN, 1 R, 1 CA	4
	7	00:56	8	12	2 RN, 1 R	3
Total		05:51	64	57	25	23

*S* surgeon, *MS* medical student, *RN* registered nurse, *R* radiologist, *CA* care administrator

*Two of the 21 surgeons were interviewed twice: one after conferences 1 and 2, and another after conferences 2 and 3

### Data Analysis

The data were analyzed according to systematic text condensation (Malterud, 2012), facilitated by the software program NVivo-11 (*NVivo Qualitative data analysis software* 2018). The unit of analysis was the transcripts of the audio recording, and the field notes and follow-up interviews for the seven conferences.

The first author iteratively listened through the audio recording for accuracy in the transcription and read the text, taking notes about the cases and how the discussions unfolded. Areas of content in the data were then formulated as preliminary themes before coding. The first author coded the text by identifying sentences and paragraphs containing information about the phenomenon while keeping the aim of the study in mind. Describing the participants’ clinical reasoning was a starting point to further analyze and understand their moral reasoning. The phenomenon was deliberately defined in an open way to be able to explore the moral reasoning close to clinicians’ everyday language. This implied not strictly separating facts and values, but rather seeing them as being intertwined, to explore moral reasoning situated in a specific context to produce meaning to answer the moral question of whether the patient should be offered surgery (de Vries & Gordijn, 2009). With the growing number of codes, those sharing similarities were assembled according to content and preliminary themes. Codes and coding groups were continuously validated against the data, moving between the parts and the whole to refine, rename and sort into subgroups, while considering the aim of the study. In the third step of the analysis, a condensation of the main coding groups was written as a meaning-oriented text, considering the aim of the study, but from a first-person perspective, representing the participants. This text facilitated the final analyses in a back-and-forth movement between the different data sources and the findings by further interpretation and reformulation of themes in a continual process of co-assessment by the authors.

To illustrate the moral reasoning that was enacted during the conference, data drawn from different transcribed conferences and field notes were utilized to write illustrative composite descriptions with direct quotations from the participants (Murchison, 2010). The illustrative descriptions contain composite extracts from multiple conferences, and data from the transcribed conferences and field notes were brought together to illustrate the moral reasoning in the discussions.

### Ethics Approval and Consent to Participate

The project was conducted in accordance with the declaration of Helsinki and received ethics approval from the Swedish Ethical Review Authority (No. 2019–04387). All participants gave informed consent prior to the case conference and written consent to the interview. The participants were informed about the aim of the study.

## Results

The moral reasoning on the vascular conferences implied the jointly anchoring of norms for a responsible balance between risk and benefit. The process implied narrating vascular suffering and general health, deliberating the benefits by scrutinizing the clinical details and assembling these elements in a reasonable proposal for further care. Additionally, moral reasoning signified amplifying perceptions and promoting transparency, promoting professional and moral learning, and supporting complex decision-making (see Table 3).

**Table 3:** Results presented by main themes and six themes

Main theme
Joint anchoring of norms balancing risk and benefit
Themes
Narrating vascular suffering and general health
Deliberating benefit by scrutinizing the clinical details
Assembling in a reasonable proposal for further care
Amplifying perceptions and promoting transparency
Promoting professional and moral learning
Supporting complex decision-making

### Narrating Vascular Suffering and General Health

The moral reasoning process emanated from a short presentation of the patient’s clinical history, providing the group with a summary description of the clinical situation by narrating the patient’s vascular suffering and their general health.

The clinical case could include ulcers and pain in the legs or growing vessels with a risk of rupture and sudden death. The patient’s other chronic diseases, such as diabetes and heart and lung conditions, followed, and, finally, a description of the patient’s general health and function in daily life. Their general health could be described by, for example, reporting walking distances and levels of outdoor and social activities, or dependence on others for practical support.

**Table:** 

Box 1 Composite descriptions of case discussion illustrating vascular suffering and striving for a balanced surgical option
The surgeons are gathered in a conference room situated in a u-shape in front of a large screen with the light slightly turned down. Surgeon A: *This is my patient. He’s a 77-year-old gentleman with severe diabetes, he’s previously amputated on the left side, he’s in a wheelchair, but he uses his right leg to move. Previous smoker, he quit a long time ago. For some weeks, he has had pain in his right foot hanging down during the night.* Looking at the screen and scrolling along the vessels. The other participants concentrate on the screen in silence. *No, there is nothing significant before we come down on the lower leg where all the three pipes are severely calcified.* Surgeon B quietly: *Typical.* Surgeon A: *My suggestion should be to do an angiography for better mapping.* The presenter is still looking at the screen: Here it’s very calcified. Surgeon C asks: *But there is some contrast there, isn’t there?* Raises to point on the screen. Surgeon A, slightly raising her voice: *Considering that he’s using his right leg for support when moving, it would be nice if we could do something. He has pain while resting and ulcers as well.* Surgeon D (sighing): *Mm, there will be an amputation in the end.* Surgeon A replies: *If we do not do anything, absolutely! I think we should puncture antegradely, keeping in mind that it’s the lower leg, if there is something we could do at all. Views on that?* Surgeon C quietly: *Just be careful.* Surgeon D: Sounds reasonable. *Next!*

Narrating about vascular suffering and general health seemed to have a twofold meaning in developing the surgeons’ understanding of the clinical situation in further exploring which care to offer the patient. First, the patient’s vascular suffering was understood in relation to the consequences of the condition for the patient’s everyday life, such as being unable to walk, being unable to sleep during the night because of pain, or being at risk of losing a leg by amputation (see Box 1). The significance of these consequences was the key foundation for determining how urgent a surgical measure was regarded to be in the coming discussion and positioned the surgeons’ perceptions before the examination of the x-ray images. A patient threatened by amputation of the leg, for example, was given more significance in trying to find a surgical solution for helping the patient when compared to a patient who was bothered by limited walking distance but not threatened with a loss of a leg.

Second, the short descriptions of the patient’s general health and comorbidities seemed to signal how much surgery the patient could tolerate without being harmed by well-meant surgical procedures. Their moral reasoning considered how surgery could inflict harm on old and frail patients, adversely affecting their health despite the good intentions of relieving the vascular suffering. Descriptions of the patient’s general health also related the vascular suffering to the consequences for their daily life, providing a context for the coming discussion to decide between different surgical options.

*If the patient walks with a walker indoors and no long distances outdoors, then we might not need to do everything we could, everything we have in our arsenal. We could choose to perform one type of surgery and think that this will probably relieve the pain so the patient could keep on walking indoors because they do not use their legs to walk for longer distances to shop outdoors. It is very important what the patient’s starting point is when you choose.* (Interview Surgeon 14, hospital 2)

Narrating about the patient’s vascular suffering and general health was preferably based on a previous clinical encounter with the patient, often in the outpatient clinic (see Box 1, 2 and 3). When the presenter of the case had met the patient, the presenter’s first-hand knowledge about the patient seemed to be given priority in the further discussion about the case. The clinical encounter was described as necessary to understand the case beyond the information in the medical records and was understood to only be presented fully if the surgeon in charge of the patient was present. The clinical encounter was important for understanding the patient’s vascular suffering and their general health, as well as a prerequisite for knowing the patient’s preferences for further surgical treatment.

*You always have to meet the patient. Glory to the records, but it is the encounter with the patient that determines how we proceed. It is then and only then that you really can appreciate what would be best for this individual. The encounter with the patient gives a feeling about whether it is reasonable to expose this individual to surgery. When you talk to patients, you sometimes get this feeling that this patient is very doubtful, this patient is not prepared to take the risks, if we force this, it will not be good. Then we need to reverse and that judgment you can only make through meeting the patient.* (Interview Surgeon 21, hospital 3).

### Deliberating Benefit by Scrutinizing the Clinical Details

From narrating about the patient’s vascular suffering and general health, the process proceeded to scrutinizing the clinical details of the case to deliberate whether surgery could relieve suffering and thereby benefit the patient. Scrutinizing the clinical details implied understanding vessel anatomy and pathology represented by constrictions and enlargements of vessels as well as taking a range of clinical details into account to explore whether any surgical options could benefit the patient (see Box 1 and 2).

**Table:** 

Box 2 Composite descriptions of case discussions illustrating the interplay between the clinical details of the case, patient’s health and risk of harm, surgical options and the need for the encounter with the patient.
The group of surgeons are gathered in the radiology department, sitting in rows in a dimmed conference room in front of two large screens mounted on the wall, where the x-ray images are displayed. The participants are making small talk in the shift between cases and one surgeon is dictating the previous discussion into a digital recorder in a low voice. The radiologist starts talking, the buzz ceases, and a concentrated silence is present while the participants watch the screens. Radiologist: *This woman has an aneurysm that has grown. If you look here and compare with the previous investigation, it’s 55 mm. Surgeon A: She hasn’t been here yet, she’s soon planned on coming to the outpatient clinic.* A phone rings and is answered by a surgeon in a low voice. Surgeon B, wondering: *We need to see here. If she’s very fit, we could go on openly now. However, if she’s weak, then shouldn’t we wait?* Surgeon C: She looks bad higher up. Surgeons are talking simultaneously, and one asks the radiologist about a measurement. Surgeon D raises her voice: *I don’t think she should be treated until she’s 60 mm.* Surgeon B: *You might be right. Nevertheless, we should at least see her.* Surgeon C: *Thera are many ways to Rome. She’s 84 years. What’s she bringing in?* Surgeon A: *Not so much. Hypertension. Vitamin deficiency. Vital, goes for walks, keeps on in her garden* (reading from the records). Surgeon D: *Not playing padel tennis?* Surgeon A: *No!* Laughs. Surgeon C to the radiologist: *The neck is quite long?* Radiologist answering: 26. Surgeon C: *So, it’s treatable.*

Scrutinizing the clinical details to understand the clinical scenario could mean asking clarifying questions about the vessel’s measurements and course, as well as changes over time after the patient had undergone repeated investigations. These clarifications substantiated the deliberation about whether a surgical procedure could be of benefit. The presence of enlarged vessels generated discussions about whether the patient would benefit from surgical treatment to prevent rupture and sudden death. The surgeons described general recommendations for surgery of enlarged vessels, however, these were seen as being general and requiring further considerations about the surgical complexity of the concrete case to indicate more specific risks of complications and any potential harm to judge whether it was right and responsible to offer the patient surgical treatment. Descriptions of serious calcifications in the large vessels could imply a greater risk of stroke during an operation and thereby serious harm by surgery (see Box 2). The moral reasoning concerned how the complexity of the procedure, and with it a greater risk of harm, implied demands of greater risk or more vascular suffering presented by the disease itself to justify offering the patient surgical treatment while striving for balance between the benefits and harms.

*Then we would have to disconnect above the kidney arteries, but the vessel looks bad and then we know that this is a more complex procedure. Well, women have an indication for surgery at 55, but that is arbitrary. When it gets more complex, then we want them to be larger, because the risk is greater with the procedure. Then we are glad we do not go on to that patient because it can turn out very bad, the greater risk, the greater benefit, all the time.* (Interview Surgeon 17, hospital 3)

While examining the x-ray images, the participants repeatedly returned to discussing the significance of the vascular suffering, asking questions about walking distance, night pain or extension of the leg ulcers, appearing to link the vessel anatomy on the screen in front of them to the significance of the patient’s vascular suffering. The surgeons searched medical journals for information during their discussion, but foremost they seemed to rely on the surgeon who had met the patient in the outpatient clinic, giving authority to the encounter with the patient (see Box 2 and 3). The patient’s general health and comorbidities informed the surgeons about what surgical measure the patient could tolerate, keeping the risk of harm in mind, and they asked clarifying questions about the degree of, for example, heart and lung failure.

Returning to the clinical history, the surgeons could reason about whether the patient might be more limited in life by their other health issues, such as chronic back pain, than by their constricted vessels in the legs. They wondered whether it was reasonable to expose the patient to potential harm to improve the circulation in the legs to achieve increased walking distance. Recurrent questions regarded whether the patient was suffering from constrictions of the vessels in the legs threatened by amputation, as this condition had a greater significance for the patient compared to pain when walking and did not represent a threat of losing the leg.

During the deliberation, the participants repeatedly returned to discussing whether a surgical option could benefit the patient’s problem, such as whether the leg ulcer would heal. This moral reasoning concerned their responsibility of not exposing the patient to surgical procedures of low value or without actual benefit to the patient. They reasoned about having different surgical options for treatment that varied in terms of placing strain on the patient, as well as their effectiveness on the vascular suffering, and they deliberated to find a reasonable surgical option that was not too burdensome and would not inflict harm, while at the same time benefitting the patient.

*He is not a candidate for a large procedure; he has COPD and five other serious diseases as well. Therefore, it was my suggestion to do a limited procedure in the groin that is a quite small intervention. Then (name of colleague) suggested using the shock wave balloon, which is a new balloon that cracks these hard calcifications and that is a smaller intervention. We said we should do that, we shall not put on a spectacle, as little as necessary to save the leg. Because he actually had a black toe.* (Interview Surgeon 7, hospital 1).

### Assembling in a Reasonable Proposal for Further Care

From deliberating about the benefits by scrutinizing the clinical details, the discussions were rounded off by assembling these considerations in a reasonable proposal for further care. Being reasonable implied striving for balance between the benefit of the surgical option and not inflicting unwarranted harm by creating surgical complications. Reasonableness was repeatedly challenged by opinions about whether the surgical option was balanced and responsible.

The final decisions could be to perform new examinations or to plan for a prompt operation. The decision could also be more open-ended, for example, describing the need for new encounters with the patient in the outpatient clinic, as the necessary knowledge about the patient’s complaints, general health and wishes for further care could be missing and a new clinical encounter was necessary (see Box 2 and 3).

**Table:** 

Box 3 Composite descriptions of case discussion illustrating vascular suffering and health, encountered authority, amplifying perspectives, and levelling of perceptions.
The surgeons are gathered around a long conference table in front of two large screens with the radiologist sitting in the back of the room. The x-ray images are displayed on two large screens on the wall with the light slightly dimmed and the participants interchangeably looking at the screen and at each other across the conference table during the discussion.
Surgeon A: This is from (name of a colleague). *The woman has an ulcer on her right leg, severe resting pain and sleeps with the leg hanging down on the floor. She has hypertension, diabetes, she’s on insulin, atrial fibrillation and on warfarin. The problem is the right.* The radiologist follows, describing the vessel anatomy. It is quiet in the room; everyone concentrates on the screen. The radiologist sums up: *Stenosis in the proximal SFA and profunda, quite a long stenosis in the abductors and finally occluded in poplitea.* Surgeon B: *This case needs some considerations.* Surgeon C suggests: *Angiography, write a referral.* Surgeon B again: *We could also consider primary amputation. 87 years old, multilevel disease, you don’t want to stent, we could not do a bypass, if we rechannelize in several places, patency will be bad in the long run.* Surgeon C hesitatingly: *Well, yes. A phone is ringing. I have to go to the operating ward.* A surgeon leaves the room. Surgeon A: *The extent of the ulcer determines quite a lot. Surgeon B: We don’t know, it is (name of surgeon) who has seen her.* Turning to the computer and reads from the records: *3 mm large ulcer.* Surgeon C: *Then I think it’s angiography. Couldn’t it be sufficient that she gets some more circulation in her profunda?* Surgeon A: *I’ll write the referral with priority.* Surgeon B: *Yeah, and add something about other necessary measures. Sedation?* Surgeon B continues reading from the records: *Anxiety when she’s lying down. Maybe dyspnoeic as well.* Surgeon C: *Does it say anything about whether she lives at home or in an institution?* Surgeon B continues reading: *Homecare, support with medication. She seems to live by herself.* Surgeon B: *Well. Feels like it is about gritting life’s downhill journey, in spite of what we do.* Surgeon A: *We have to assume that (name of colleague) had the opinion that it was worth doing something at all.* Surgeon C: *Well, shouldn’t we take her back to the outpatient clinic to do a new assessment?* Surgeon A: *Yeah, I think we need to reason with her again.*

Questioning reasonableness could comprise patients suffering from critical ischemia, with pain and ulcers in the legs generating concerns about whether vascular surgery should be offered at all or whether, primarily, amputation was in the best interests of the patient. Questions concerned whether a surgical procedure could provide a reasonable chance to save the leg, heal the ulcers and relieve the vascular suffering. Balancing implied, on one hand, the patient’s severe suffering and loss of general health, and, on the other hand, the implications of losing a leg for the patient’s mobility and independence. Reasoning concerned the potential for overtreatment and adopting an overly positive attitude to the results of surgery and a responsibility to question whether the suggested surgery would benefit the patient. However, considerations about the under-treatment of constricted vessels in the legs were also present, as was having a too-cautious attitude, being afraid of adverse results, and thereby potentially leading patients to not being offered the necessary vascular care to avoid amputation.

*I do not think it is in the patient’s best interest to keep poking in the vessels. If it is an advanced condition but considering their health and life in general, they might not have any benefit of that. Their problem might be bad memory, loneliness, and pain in a hip. Neither I nor anybody else wants people to suffer, but sometimes our struggle to be good people and good doctors is not in the patient’s best interest. It is better to amputate so they can get rid of their pain at once.* (Interview Surgeon 1, hospital 1)

Another recurrent challenge concerned reasonableness in offering prophylactic surgery for enlarged vessels, aiming to prevent sudden death. The reasoning included advanced technical details about what could be done, as well as consideration of what the heart, lungs and kidneys could endure with the risk of complications and harm. The completing of the deliberations included questioning whether offering a prophylactic measure to prolong the patient’s life and avoid death by rupture was reasonable at all. Experiences were present that the technicalities about what could surgically be accomplished had the potential to overshadow the question of reasonableness.

*I rarely experience vascular patients to be self-evident; absolutely, we shall operate! With many patients, we have this weighing, shall we, shall we not? They are very diseased from the beginning. There are always advantages and disadvantages. When we choose to operate it is because the advantages outweigh a little bit more, and then it turns out to be reasonable to operate or reasonable not to operate, to refrain from surgery.* (Interview Surgeon 15, hospital 2).

### Amplifying Perceptions and Promoting Transparency

Answering the moral question of what surgical care the patient should be offered appeared as the main purpose of the conference and good surgical care could be mediated by amplifying perceptions and promoting transparency during the deliberation.

Surgeons expressed the case conference as crucial for securing and developing better care for the patient. A reason given for the particular importance of discussing together by amplifying the perceptions of the case was lack of robust evidence and that the surgical options included a large range of different surgical possibilities all implying risk of harm. The perceptions could imply the variety of surgical solutions and the aim of exploring these details together was to strive for a reasonable balance between risk and benefit. Surgeons described how they saw the quality of the surgical care as being embodied in the clinical and technical details of the case (see Box 3).

However, amplifying perceptions for promoting quality also implied an element of transparency to function as a form of control. A need to modify different perceptions about what was considered a balanced surgical offer was present, as they were operating on each other’s patients. Even if continuity was desirable for the patient as well as the surgeons, this could not always be accomplished when providing timely care to patients. Surgeons described how carrying out colleagues’ decisions implying a need for mutual adjustment of their understanding of what represents a balanced surgical option. Considerations of the patient’s vulnerability being exposed to the power of the surgeon’s decision-making practices entailed a responsibility for transparency and control to ensure the safety of the patient.

*Above all, more opinions. Have I thought right? A breaking point and sometimes very wise opinions. Have you thought about this? The patient is like this or like that, is this really the case? It is a quality control, which, I think, is very important. Maybe I am more conservative, maybe you are less conservative, and there is a grey scale. How am I to be controlled, who is going to look after what we are doing here?* (Interview Surgeon 11, hospital 2).

### Promoting Professional and Moral Learning

The deliberations about what could surgically be done, as well as the moral questions of what should be offered to the patient implied promoting professional and moral learning between the participants in the case conference.

The surgeons described how they discussed many cases within a short period of time, deliberating a range of surgical options. For younger surgeons, these discussions could be essential for demonstrating how they should proceed in framing further care for the patient. Even more experienced surgeons described the value of continuous professional learning by exposing themselves to a greater volume of cases discussing the various surgical options.

Participating in the moral reasoning process also enacted a form of learning which could be understood as a type of moral learning. The multiple perspectives, arguments and complexities described in their reasoning was conveyed as being important for learning not only what surgically could be achieved, but also what morally ought to be offered to the patient. Surgeons described large variations in how vascular surgery is performed nationally and internationally, and the conference held an important significance in negotiating how they should navigate in the responsibilities of offering the patient a balanced of surgical offer, signifying professional as well as moral learning.

The different opinions gathered in the discussions enacted moral learning by promoting the ability to adjust one’s own perceptions about what was responsible to offer the patient. They described how they could have different opinions of what was reasonable to do for very old people, or at which age it was reasonable to perform prophylactic and potentially harmful surgery, striving for a reasonable balance between risk and benefit. Different views were also expressed about how much surgery should be attempted to rescue legs from amputation. These negotiations for establishing a responsible and balanced surgical option were mediated through a process of moral learning.

*It is precisely this ethical compass, that you calibrate the compass at every case conference. “Is this worth it? Are we doing any good? How do I assess the risk of complication?” After all, we bring together several individuals, you have a greater insight into literature, and there are several brains that have read articles.* (Interview Surgeon 19, hospital 3).

### Supporting Complex Decision-Making

The case discussions also signified the need for support in complex decision-making, which signified a sense of confidence for the surgeons. They described their decision-making processes as being difficult, entailing the balancing of a wide spectrum of perspectives and details that should be gathered into a whole, creating sound and responsible options for the patient. Discussing complex cases with colleagues implied engaging in effective arguments, finding surgical options, and questioning reasonableness to illuminate the case, which could provide the surgeons with a sense of confidence in carrying out these complex decisions.

However, this sense of support did not seem to imply the sharing of the moral responsibility for the final decisions with the whole team. Discussing complex decisions during the conference could imply experiencing a sense of confidence in the decision-making process, but the moral responsibility could not be shared beyond the surgeon in charge of the patient. Instead, the final responsibility for an operation rested with the operating surgeon. They described being obliged to interrupt planned procedures if they could not support them morally. This was as a delicate matter and substantiated the need for anchoring decisions about coming procedures in the group, especially when they were complex, or the surgeons were doubtful.

*I have a great need, even though I have worked here for a long time, I think it is very good to get other people’s views on the patient. Our decision-making is difficult. It is still the way that I can feel – oh, there I was completely wrong. After talking to some colleagues, I see it in a completely different way. It is clear that it is better to treat this patient or do not treat. A well-considered and good decision, that you get after discussing with others.* (Interview Surgeon 18, hospital 3)

The moral reasoning during the conferences repeatedly reflected on the risk of inflicting harm on patients. Harming patients seemed to place the surgeons in an exposed situation where the case discussion could function as a form of support, even without lowering the moral responsibility. The surgeons reflected on the importance of the climate of the discussions. A prerequisite for the supporting function seemed to be experiencing the climate as being open and permissive, where different perceptions, as well as questioning, were encouraged by the members. However, the surgeons also described experiences of informal leadership and power, where clinical experience gave legitimate influence, but where informal power was present as well.

*It is true that some people are very strong informal leaders and their opinions on what to do carry a lot of weight. Whereas if another person had said the same, it might have been ignored. We never get away from these group dynamics and it is difficult to manage. So, one can hope that the decisions will be as correct as possible regardless of who is there, but it is absolutely the case that some people’s opinions carry more weight. Sometimes it is right because you have more experience, but sometimes actually just purely from the informal leadership.* (Interview Surgeon 20, hospital 3).

## Discussion

The moral reasoning process implied a jointly anchoring of norms for a responsible balance between risk and benefit by narrating the patient’s vascular suffering and general health, deliberating what could benefit the patient by scrutinizing the clinical details and assembling in a reasonable treatment proposal to offer to the patient. Additionally, moral reasoning signified amplifying perceptions and promoting transparency, promoting professional and moral learning, and supporting complex decision-making.

The moral reasoning during the conferences, as experienced by the vascular surgeons, was embedded in a discourse where the moral and clinical reasoning were tightly interwoven in the surgeons’ medical language and included, to a lesser extent, the patient’s wishes for care. It could be conveyed that the surgeons seemed to be paternalistic in framing care according to what they considered beneficial for the patient, suggesting a lack of involvement of the patient in the decision-making. Two previous qualitative studies of MDTs within cancer care show similar results, describing how clinicians who make decisions based on medical information alone lack the inclusion of patient perspectives (Hahlweg et al., 2015; Hamilton et al., 2016). However, we have previously studied these vascular surgeons’ individual moral reasoning for outpatients, and our results showed how the encounter with the patient substantiated their moral reasoning by shifting the perspective from the vessels to the whole person, implying that they did explore the patient’s wishes for care as well as their vascular suffering and general health (Heidenreich et al., 2023). The present study describes vascular surgeons’ needs to explore the surgical options in a team of surgeons by amplifying and adjusting perceptions, thereby capturing the complexity of the practice, and can be interpreted as an exploration of what could be offered to the patient. The scope of the case discussion is to understand the vascular disease, deliberating on what is reasonable and responsible to offer the patient. The surgeons in charge of the patient thereafter went back to the patient for a discussion about what ought to be done.

Recurrently, the surgeons paid attention to information gained from the clinical encounter with the patient, giving the surgeon in charge of the patient a form of encountered authority in the reasoning process. The information comprised the patient’s vascular suffering, general health, daily life as well as their wishes for care. A lack of knowledge from the clinical encounter, where none of the surgeons attending the conference had met the patient, impaired the reasoning process, and rendered it only possible to proceed on a more general level, which often concluded with a need to arrange new encounters with the patient. Encountered authority has been described in a previous study of MDT in cancer care as a source of authority given substantial weight when considering different treatment pathways for the patient (Dew et al., 2015). The weight of the clinical encounter in answering what ought to be offered to the patient was also seen in our outpatient study (Heidenreich et al., 2023). It might seem obvious that the closeness that is provided in encountering the patient provides substantial understanding for the framing of further surgical care. However, the moral reasoning in the present study signified reasoning morally together based on the closeness obtained from the encountered authority intertwined with reasoning based on the other participants having some distance from the patient. The value of the group members’ different perspectives in allowing adjustments of perceptions as well as transparency implied a need for distance as well as closeness in the decision-making process. Patients are vulnerable to professionals’ power, and the conference could represent a review beyond the relationship between a surgeon and a patient and thus the significance of this transparency was apparent.

The overarching conflict of values in the surgeons’ moral reasoning were unsurprisingly found to be between risk and benefit, displaying a conflict between the principle of non-maleficence and beneficence (Beauchamp & Childress, 2013). However, what the study adds empirical knowledge about, is how the surgeons seemed to handle the conflicting values during their case conferences. In their moral reasoning processes, they tried to achieve a reasonable balance between risk and benefit by exploring the clinical details of the case by concretization and balancing facts about the case. The surgeons seemed to explore the clinical details of the unique patient case to balance the conflicts of values and move forward in the decision-making process. They sought facts about the case to solve the conflict of values through a process of concretization and specification of the case. These details and facts could be concrete measurements, such as the width of the aorta and degree of kidney failure but also comprised the degree of suffering and limitations in the patient’s daily life. Some facts were found in the patient’s records, but necessary understandings of the case were generated in the face-to-face encounter with the patient in the outpatient clinic. Discussing cases together anchored norms for the balance between risk and benefit in the concrete case by generating understandings through the amplifying of perceptions and promotion of transparency and assembling these considerations in a reasonable plan to offer the patient.

Enabling opportunities to support surgeons in complex decision-making processes for their own sense of confidence indicates an interplay between ethical and existential issues in vascular surgeons’ daily care of patients. The risk of inflicting harm on patients was recurrently present during the moral reasoning process, which also seemed to place the surgeons in a vulnerable position, needing support for their assurance in performing procedures with a potential risk of harm. Generally, all surgical care inflicts harm on patients, and cure through harm is a general prerequisite of surgical practice. However, the extent to which the inflicting of harm was regarded as being balanced was an ongoing element of the moral reasoning process, negotiating the responsibility of protecting the patient from unwarranted harm and concurrently striving for the relieving of suffering.

Existential issues encountered by thoracic surgeons and cardiologist have been previously explored, describing a sense of vulnerability when dealing with responsibility and mistakes, and indicating the need for support (Aase et al., 2008). A distinct surgical ethics has also been described on a theoretical level, where the categories of rescue, proximity, ordeal, aftermath and presence outline the surgical encounter as having inherent relational, ethical and existential elements (Little, 2002). The surgeons’ experiences of the case conference as being supportive could be understood as being a means for dealing with their own vulnerability when making decisions with the potential for inflicting unwarranted harm on patients seeming to be an inherent existential issue in their care practices. Discussing these issues together, primarily for promoting better care of the patient, could promote an increased sense of confidence in complex decision-making. However, the responsibility for the patient’s outcome seemed to ultimately rest on the operating surgeon and, despite this experienced support, this aspect of moral responsibility did not seem to be shared. The experiences of the importance of creating a climate of permissiveness to promote professional and moral learning and provide support was present, and team-culture has been described as important for the quality of MDT meetings in cancer care (Walraven et al., 2022). Bullying, undermining behaviour and harassment are also reported as being prevalent within surgical practice, making a major impact on the victims (Halim & Riding, 2018). Our observations did not show any evidence of such behaviours, probably due to the selection of the units. However, this underscores the importance of an ethical climate as a prerequisite for moral reasoning, and the necessity of clinical ethics support that is perceived as relevant by clinicians.

Considering the tightly interwoven nature of moral and clinical reasoning in vascular surgery, clinical ethics support should be carefully adapted to the specific clinical context. Previous research suggests that surgeons tend to perceive such support as less relevant when it is disconnected from the realities of clinical care (Orlowski et al., 2006). Building on our findings, we suggest that clinical ethics support may be more meaningful to vascular surgeons if it is integrated into the care of concrete patients and framed within a consequentialist ethical framework. As our study highlights the role of case conferences in fostering professional and moral learning, the ethics support service might be adapted to these existing forums to enhance this learning, rather than introducing entirely new support structures. However, further research is needed to explore how clinical ethics support can be structured to align with the moral reasoning and clinical realities of vascular surgeons.

## Methodological Considerations

Of the seven invited hospitals, only three agreed to participate. The units who declined provided lack of time and interest, as well as research ethics concerns about sensitive patient information exposed during the conferences, as reasons. It could be argued that the participating units might have a greater interest in ethics and may have promoted a more permissive climate in their conferences, thus reducing the transferability of the results. However, including three different participating units also provided a level of social diversity, which strengthens the validity of the results.

Surgeons being approached for follow-up interviews were selected based on their participation in the previous conference discussion. No participants declined the interview, but the procedure excluded surgeons who were more reticent during the conferences and, consequently, constitutes a weakness as elements of moral reasoning may have been missed. As displayed in Table 2 two surgeons was interviewed twice after two different conferences, so the purposeful sampling strategy contrived to achieve different voices in the interviews.

Combining observations with interviews rendering opportunities to explore a phenomenon that is considered difficult to capture in clinical practice. The observer and first author, as a medical clinician, possesses understanding of the medical language and surgeons’ clinical reasoning as well as the medical culture. This insider knowledge probably gave access to the field and was an important prerequisite for exploring moral reasoning embodied in the clinical language. However, this preunderstanding demanded a process of making the familiar strange, adopting openness and curiosity increasing reflexivity and can also be assumed to be further reduced by the other authors’ critical reflections during the research process (Patton, 2004).

One researcher carried out the observations and, with the phenomenon of moral reasoning at hand, the focus was placed on the verbal utterances. To a lesser degree, the observations captured body language and group dynamics that may have been enhanced by including two observers or video recordings. Being present could have had an impact on the interactions in the group, perhaps causing them to behave in a more socially desirable way. However, the conferences were a part of their weekly routine as an accepted part of their practice, which could have reduced the observer’s impact on the group. Moral reasoning is probably a part of a team’s moral culture that to some degree is local and contextual. However, the ethical issues concerning the framing of responsible care for their vascular patients probably have similarities outside this contextual frame, giving the results informative significance for other vascular units.

## Conclusion

Vascular surgeons moral reasoning during case conferences was a process of jointly anchoring norms for balancing patient risk and benefit. The process of moral reasoning on the case conferences highlights the dual authority between the conference’s deliberations of good surgical care and the authority from the surgeons’ clinical encounter with the patient. The encountered authority embodies the patient´s voice as well as a clinical based understanding of suffering, health, and risk of harm. This encountered authority, faces the authority of the conference by facilitating more perceptions about the case and promoting transparency to assembling in an offer to the patient. In the process of moral reasoning, clinical, ethical, and existential issues are embodied, and this complexity demands conditions for deliberating the framing of patients’ care by providing well adapted clinical ethical support targeted to existing case conferences.

## Data Availability

The datasets generated and analyzed during the current study are not publicly available due to the current Swedish ethical legislation and European Union GDPR act.
